# Characterisation of Nanoclay and Spelt Husk Microfiller-Modified Polypropylene Composites

**DOI:** 10.3390/polym14204332

**Published:** 2022-10-14

**Authors:** Madara Žiganova, Remo Merijs-Meri, Jānis Zicāns, Tatjana Ivanova, Ivan Bochkov, Mārtiņš Kalniņš, Andrzej K. Błędzki, Paulius P. Danilovas

**Affiliations:** 1 Institute of Polymer Materials, Faculty of Materials Science and Applied Chemistry, Riga Technical University, 3 Paula Valdena Street, LV-1048 Riga, Latvia; 2 Institute of Materials Science, West Pomeranian University of Technology, Department of Materials Engineering, 19 Piastów Avenue, 70310 Szczecin, Poland; 3 Polymer Competence Center, Gradauciznos k. 7, LT-60430 Raseiniu R., Lithuania

**Keywords:** reinforcements, mechanical properties, thermal properties, biobased polymer composites, polymer modification

## Abstract

Current research is devoted to the investigation of spelt husk (SH) and nanoclay-modified compatibilised polypropylene (PP) binary and ternary composites for injection-moulding applications. PP composites were obtained using twin-screw extrusion. The content of mechanically milled SH microfiller with aspect ratio within 2 and 6 was fixed at 40 wt.%, whereas the amount of nanoclay functional filler in the polypropylene matrix was changed in the range from 0.5 to 5 wt.%. Nanoclay filler was introduced in the polypropylene matrix either in the form of nanoclay powder (C) or as a masterbatch (M). Regular distribution of the clay nanofiller within the polymer matrix has been observed, disregarding its form and concentration. The effects of the individual or combined addition of SH microreinforcement and nanoclay fillers on the rheological, mechanical, calorimetric, and thermal properties of the developed PP composites were investigated. It is revealed that the addition of the nanoclay fillers insignificantly influences the viscosity of both PP nanocomposites and hybrid composites with SH. Additionally, for PP nanocomposites, remarkable increases in tensile and flexural modules and strength are observed by maintaining considerable ultimate deformations, mainly in the case of M-containing systems. Concomitantly, because of the addition of the nanoclay filler, the improvement in thermal stability of PP nanocomposites and PP hybrid composites with SH is observed. As a result of SH addition, considerable increases in tensile and flexural modules are also observed. Results of the research demonstrate the potential of the use of natural materials (agricultural residues and clay minerals) for the development of PP composites with increased stiffness and thermal properties.

## 1. Introduction

The current demand for environmentally friendly materials makes researchers and commercialists focus on alternate options to synthetic materials, particularly polymers, which are commonly derived from fossil sources. Over past decades, polymers have replaced many conventional materials such as metals, glass, and wood in various applications starting from low-cost consumer articles and ending with high-tech customized products for transport, construction, and energy sectors. In transport and construction sector applications, polymers are often used as matrices of composite materials that meet the demand for high-performance materials with increased mechanical, thermal, and other valuable properties. Increased use of polymers and polymer-based materials in almost every branch of the national economy has led to a generation of a great amount of postconsumer polymer waste, particularly originating from sources of short-term applications such as packaging and disposables. Nevertheless, the use of polymer-based products in durable construction and transport applications represents a delayed threat of generating polymer waste, as these durable materials tend to appear in the waste stream several years after the start of their application. Consequently, in the design of new products, several aspects should be taken into account, meeting the demands of environmental friendliness, cost efficiency, technological prevalence over the existing materials, and market viability. Considering the case of durable products, increased environmental friendliness may be ensured by developing polymer-based composites, which are reinforced with natural fibres, while only limited biodegradability in a specified environment is accepted in order not to compromise the longevity of the product. Cost efficiency is usually ensured by developing polymer composites based on low-cost recycled or waste products, such as postconsumer polymers, biomass waste, etc. Technological prevalence is achieved by ensuring sufficient interaction between the components of the developed polymer composite as well as by modifying the polymer matrix with synergistically interacting and mutually completing ingredients. Market viability, in turn, is largely dependent on environmental awareness and the customers’ willingness to pay, even if the proposed product is more expensive than its environmentally less friendly counterpart. These aspects were based on the current research on the compatibilized polypropylene (PP)-based hybrid composites reinforced with grain husk-derived lignocellulosic microfiller and nanoclay. According to the recent review by Yashas Gowda et al. [1], PP is one of the most widely used matrices for the development of natural-fibre-reinforced polymer composites because of its recyclability, moderate processing temperatures, as well as relatively high mechanical properties. Considerable increase in mechanical properties, particularly modulus, tensile, and flexural strength, has been reported, especially in the case of compatibilized systems [2]. Various approaches have been used to increase compatibility between nonpolar PP matrix and highly polar natural microfibres, including fibre pretreatment by physical [3,4] or chemical [5,6] means, as well as the addition of a separate compatibiliser [7]. Although various chemical compatibilisers have been used to increase interfacial adhesion between PP matrix and natural fibres, with respect to cost/performance ratio, the maleic anhydride groups containing compounds have shown the greatest efficiency [8]. Consequently, maleic-anhydride-grafted PP copolymer has been chosen as the compatibiliser also in the current research.

With respect to natural fibre reinforcement, a great variety of lignocellulosic materials have been used to enhance the properties of PP and other polymer composites [9]. Wood flour is an abundant, low-cost lignocellulosic material that has been used for years as a reinforcement of polypropylene, primarily in an injection-moulded product market. The amount of natural fibre was chosen by considering the limitations of injection moulding. According to the literature, the amount of natural fibres in the thermoplastic polymer composites is usually limited to ca. 40–45 wt.% [10,11], while at higher reinforcements content, the quality of the injection-moulded products may be reduced, and the configuration complexity is limited. The residue of crop farming is another waste source of lignocellulosic materials. An important advantage of agricultural waste-derived fillers is their availability and annual renewability. According to estimates [12], wheat, rice and barley straw, corn stover, sorghum stalks, coconut husks, sugarcane bagasse, and pineapple and banana leaves are among the major lignocellulosic agricultural residues. Rice husks are probably the most widely used agricultural residue for the development of bio-based polymer composites because of their abundance as well as some specific properties, such as intrinsic SiO_2_ content, which lead to reduced moisture sensitivity [12,13]. Bledzki et al. [14,15] considered rice, rye, and wheat husks as potential alternative fillers for softwood fibre in bio-based PP composites, demonstrating better deformability and increased impact strength. Similarly, in our previous research, it was demonstrated that the addition of either mechanically milled or steam-explosion-treated oat or spelt husk flat microfibres allows more than a twofold increase in the modulus of elasticity, as well as an improvement in tensile and flexural strength of PP composites in comparison with the neat polymer matrix. In contrast, the ultimate elongation of the grain husk microfiller-containing systems was larger than that of the wood flour filler-modified counterpart [16].

Natural-fibre-reinforced polymer composites unfortunately also have certain drawbacks, for example, moisture and thermal sensitivity. Consequently, to reduce the negative effects of moisture uptake and thermal degradation during processing, adding an extra additive should be considered. Layered silicates, such as montmorillonite nanoclay, offer a high surface-to-volume area, a high aspect ratio of monoplatelets (as high as 448 in the case of montmorillonite [17]), high stiffness (178 GPa [18]), and impermeability to gases and solvent vapours [19]. This makes montmorillonite nanoclay suitable for increasing mechanical and thermal barriers and other properties of polymers. In order to increase compatibility between polar montmorillonite nanoclay particles and nonpolar polypropylene matrix, a variety of approaches have been used, based on both organofunctionalization of the mineral filler and introduction of customized compatibiliser in the composite. Functionalisation of montmorillonite particles is based on ion exchange reaction to replace originally occurring inorganic cations in the interlaminar space of the silicate mineral with low- or high-molecular organic surfactants, leading to an increase in the distance between the clay monolayers. This aids penetration of polymer chains into the clay interlaminar space, facilitating further separation of nanoplatelets and yielding intercalated or even exfoliated structures under applied shear stresses during the manufacturing of polymer nanocomposites. In addition to the organomodification of nanoclay, customized compatibiliser is another common approach for improving dispersibility and increasing interaction between montmorillonite nanoclay and polypropylene matrix. Although various interfacial agents have been used, including silane (mostly trialkoxysilanes) [20], titanate, and zirconate coupling agents [21,22], maleic-anhydride-grafted polypropylene still demonstrates remarkable technological efficiency at reasonable costs.

As previously mentioned, increased attention has been paid recently to the development of environmentally friendly hybrid composites containing polypropylene matrix, lignocellulosic reinforcement, and clay nanofiller. In recent comprehensive reviews on natural-fibre and nanoclay-reinforced polymer hybrid composites [19,23], modifications of polypropylene simultaneously with almond shell flour and organically modified montmorillonite [24], wood fibre and nanoclay [25], poplar sawdust and nanoclay [26], wood flour and nanoclay [27,28], pine cone fibres and nanoclay [29], reed fibres and nanoclay [30], bagasse and nanoclay [31], coir fibre, wood fibre, and montmorillonite [32], and carbon fibres, glass fibres, montmorillonite and wood fibre [33] have been demonstrated. In these research works, an increase in mechanical (particularly stiffness) and a decrease in water sensitivity, along with the addition of nanoclay, are generally demonstrated. At the same time, the increase in the mentioned properties in some cases is rather moderate; additionally, a significant increase in thermal properties is observed to a considerably lesser extent. Analysis of rheological properties of natural fibre and nanoclay-modified polypropylene hybrid composites is also not sufficiently performed. Consequently, only a limited number of investigations exists related to grain husk microfillers and nanoclay-modified polypropylene hybrid composites. Therefore, the current research analyzes how the addition of locally obtained spelt husk microreinforcements, as well as montmorillonite nanoclay, affects the rheological, thermal, and mechanical properties of polypropylene. It is expected that the use of a simple melt mixing method for the development of PP composites with SH and its hybrid composites with nanoclay will promote the utilization of agricultural residues (grain husk) for the development of value-added composites with improved stiffness and thermal stability. Furthermore, it is expected that this approach could be further extended to recycled polyolefin matrices.

## 2. Materials and Methods

### 2.1. Materials

**Figure 1:**
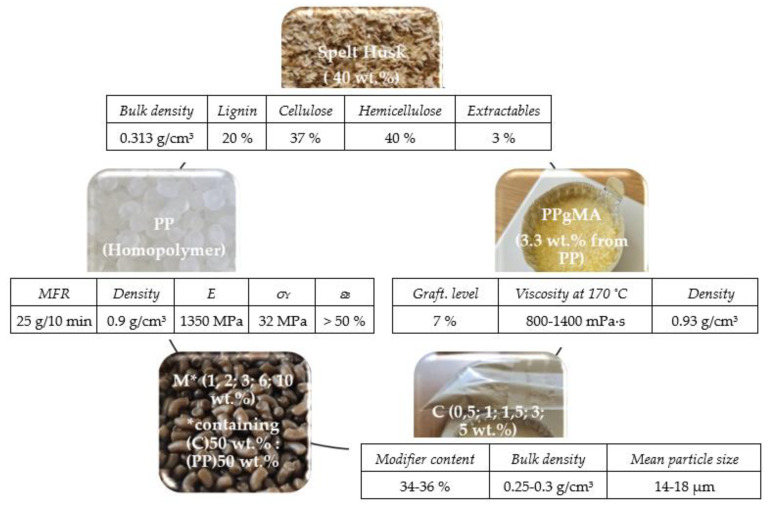
Schematic presentation of raw materials used for research.

Injection-moulding-grade polypropylene homopolymer (PP, *Moplen* HP400R, Bassel Orlen Co., Płock, Warszaw, Poland) was used as a matrix. This homopolymer exhibits good stiffness and high fluidity (MFR = 25 (230 °C/2.16 kg), necessary for injection-moulding applications. Polypropylene-graft-maleic anhydride (PPgMA, Clariant Co., Licocene PP MA 6452, Muttenz, Switzerland) in the amount of 3.3 wt.% in relation to the matrix was used as a coupling agent between the nonpolar matrix and polar lignocellulosic fibres. Spelt husks (SH, Amilina AB Co., Panevėžys, Lithuania) microfiller used as lignocellulosic reinforcement was obtained by mechanical milling. Two types of organoclays containing fillers, neat nanoclay (C, Nanomer I.44P, Nanocor Co., Hoffman Estates, IL, USA ), and organoclay masterbatch (M, Masterbatch I.44P, Nanocor Co., Hoffman Estates, IL, USA), were used as co-modifiers. A visual summary of all the raw materials is shown in Figure 1, along with their main physical characteristics.

### 2.2. Preparation of the Composites

Before obtaining the bio-based composites, raw SH was mechanically sieved through 3 mm and 2 mm sieves to remove coarse impurities on the one hand and dust and grain residues on the other hand. Thus, isolated SH were ground at 700 rpm using Retsch (Retsch GmbH, Haan, Germany) cutting mill SM300 with a 0.25 mm sieve. Sieve analysis of the milled SH was performed using pneumatic sieve shaker LPzB-2e (Multiserv-Morek Jan Morek, Brzeźnica, Poland) equipped with a number of sieves within the following size range: 0.05 mm–1.0 mm. Considering that in the case of injection moulding of the investigated composites considerable rise in viscosity is expected, the amount of SH in PP composites was fixed to 40 wt.%, not exceeding the 45 wt.% limit accepted by the industry [10,11]. In the case of M and C, the maximum concentration in the PP matrix was limited to 5 wt.% in accordance with the manufacturer’s specifications. It is known that at higher nanofiller content, increased agglomeration may be observed.

Before further melt processing, the milled SH microfillers were dried overnight in an oven at 60 °C. The organoclay containing fillers were dried at 60 °C in a vacuum oven for 3–4 h.

SH microfiller and nanostructured organoclays at certain ratios, as shown in Table 1, were compounded with PP matrix in the presence of PPgMA compatibiliser using Thermo Electron Corporation (now Thermo Fischer Scientific Inc., Waltham, MA, USA) twin-screw co-rotating extruder PRISM TSE 16 at the screw rotation speed of 40 rpm and the following temperature profile over the 5 heating zones: 170–175–180–185–190 (die) °C. The extrudates obtained at the above-mentioned compounding conditions were cooled in a water bath and pelletized. Thus, obtained pellets were dried in a vacuum oven for 4 h under 80 °C before further processing by injection moulding using an injection-moulding machine Minijector 55 (Miniature Plastic Molding, Farmington Hills, MI, USA) at the following temperatures of the barrel heating zones: 165–190–200 (die) °C. Dimensions of injection-moulded flexural test bars were 80 mm × 10 mm × 4 mm, in accordance with EN ISO 178, whereas the dimensions of tensile dog-bone test specimens were in accordance with EN ISO 527-2 (Type 5A).

### 2.3. Characterization of the Composites

#### 2.3.1. Light Microscopy

Inspection of the isolated fractions of sieve analysis of SH was performed by LEICA optical microscope Leica DM RM (Leica Microsystems GmbH, Wetzlar, Germany) at 5× magnification by performing at least 20 length-to-width measurements per individual microfiller particle. For shape analysis of SH microparticles open-source image analysis tool Fiji (Fiji is just ImageJ, ImageJ, Eliceiri/LOCI group at the University of Wisconsin-Madison, Jug group at Human Technopole in Milan, and Tomancak lab at the MPI-CBG in Dresden) was used.

#### 2.3.2. Scanning Electron Microscopic Analysis (SEM)

Morphology was studied using Mira\LMU field emission scanning electron microscope (TESCAN a.s., Brno, Czechia), operated at 15 kV and magnification of 5000. The investigated polymer nanocomposite test specimens for SEM measurements were obtained by breaking rectangular test bars cooled in liquid nitrogen in order to obtain a brittle fracture surface. The samples were sputter-coated with gold using an Emitech K550X sputter coating unit (Quorum Technologies Ltd., Lewes, United Kingdom).

#### 2.3.3. Fourier Transform Infrared Spectroscopy (FTIR)

The spectra were obtained by Nicolet 6700 spectrometer (Thermo Fisher Scientific Inc., Waltham, MA, USA) with the Attenuated Total Reflectance (ATR) technique. All the spectra were recorded in the range 650–4000 cm^−1^ with a resolution of 4 cm^−1^.

#### 2.3.4. Rheological Tests—Capillary Rheometer

Visco-elastic properties in melt state at 190 °C were determined using Malvern capillary rheometer RH7 (Malvern Panalytical Ltd, Malvern, Worcestershire, UK). Rheological behaviour was tested at 7 different shear rates, changed stepwise from 120 s^−1^ to 12,000 s^−1^. During the experiment, shear viscosity *μ*, shear stress *τ*, and flow index *n* within the mentioned shear rate *γ*· range were determined. The procedure was repeated at least three times for each material tested.

#### 2.3.5. Differential Scanning Calorimetry

The melting and crystallization behaviour of the investigated composites was evaluated using differential scanning calorimeter DSC 1/200W (Mettler Toledo, Greifensee, Switzerland). The specimen of approximately 10 mg was sealed in an aluminium pan and subjected to the following temperature cycles: (1) heating from 25 °C to 200 °C at a rate of 10 °C/min and holding at the corresponding target temperature for 5 min, (2) cooling to 25 °C at a rate of 10 °C/min and holding at the corresponding target temperature for 5 min, followed by (3) second heating from 25 °C to 200 °C at a rate of 10 °C/min. The DSC measurements were performed underneath a nitrogen atmosphere.

Crystallinity (*X_c_*) of the PP composites and hybrid composites was calculated using the following equation:

(1) $$X_{C}=\frac{\Delta H_{c}}{\Delta H_{m}^{o}\left(1-W\right)}\times 100\%$$

where Δ*H_c_* is the melting/crystallization enthalpy of the measured composite sample, Δ*H*^0^_*m*_ is the theoretical melting enthalpy of PP crystal, assumed as 190 J/g, and *W* is the weight fraction of the additives (nanoclay and spelt husks).

#### 2.3.6. Thermogravimetric Analysis

The thermal stability of the investigated composites was analyzed using thermogravimetric analyzer TGA1/SF (Mettler Toledo, Greifensee, Switzerland). Specimens of approximately 10 mg were heated from ambient temperature to 800 °C at a heating rate of 10 °C/min under an inert atmosphere (nitrogen gas flow). The material weight loss was calculated using the original software following the ASTM D3850.

#### 2.3.7. Flexular Test

Stress–strain characteristics of the studied compositions in bending were determined using universal material testing machine BDO—FB020 TN (Zwick Roell Group, Ulm, Germany) in accordance with EN ISO 178 at a deformation speed of 1 mm/min. Flexural modulus *E_L_*, maximum flexural stress *σ_ML_*, and corresponding relative deformation *ε_ML_* were analysed. Demonstrated values represent the averaged results of the measurements performed on 10 test specimens for each type of composite material.

#### 2.3.8. Tensile Test

Tensile stress–strain characteristics of the compositions were determined at a temperature of 20 °C using material testing equipment BDO—FB020TN (Zwick Roell Group, Ulm, Germany) equipped with pneumatic grips in accordance with EN ISO 527. Type 5A test specimens were stretched at a constant deformation speed of 50 mm/min. Tensile modulus *E*, stress at break *σ_B_*, relative elongation at break *ε_B_*, yield stress *σ_y_*, and the relative elongation at yield *ε_y_* whenever appropriate were analysed. Demonstrated values represent the averaged results of the measurements performed on 10 test specimens for each type of composite material.

## 3. Results and Discussion

### 3.1. Light microscopy

**Figure 2:**
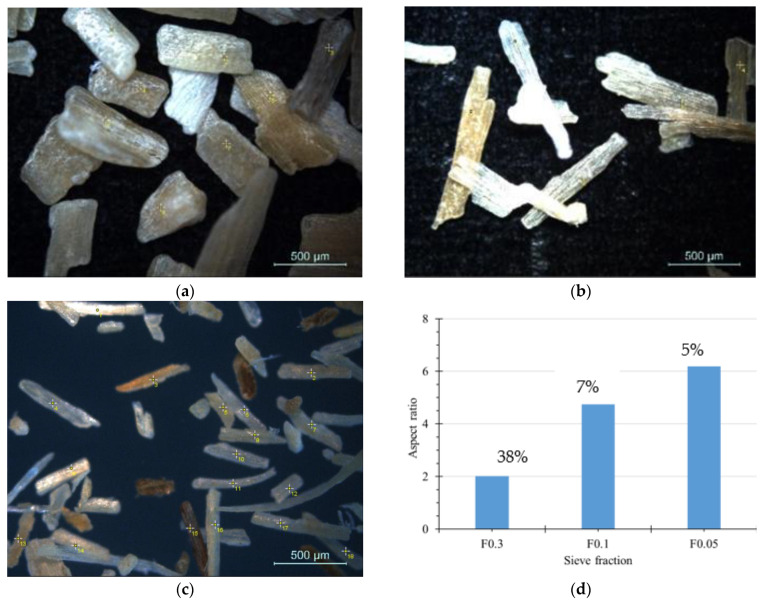
Light microscopy images of SH after sieving through 0.5 mm (**a**), 0.3 mm (**b**), and 0.1 mm (**c**); size sieves and calculated average aspect ratios of the respective sieve fractions and their per cent amounts (**d**).

Light microscopy images of the used lignocellulosic microfiller particles are demonstrated in Figure 2, representing main size fractions of SH obtained after milling.

As one can see, milling of SH flat microfibres at 700 rpm yields micron-sized reinforcements with a rather broad spectrum of particle sizes and aspect ratios. Thus, after milling average dimensions of SH flat microfibres have been considerably reduced from the initial ca. 10 mm in length and ca. 3 mm in width. Although lateral and axial dimensions of the used SH microparticles are reduced because of milling, it is revealed that the microfibrillar morphology of SH microfibre reinforcement is maintained at each of the isolated fractions. Along with changes in its dimensions, considerable changes in the aspect ratio of the used SH flat microfibres are observed. Considering the obtained amounts of the separate sieve fractions, it was concluded that the further development of PP composites uses SH microfibres in size range of 0.05–0.3, building up more than 50% of the total amount of the sieved material.

### 3.2. SEM

**Figure 3:**
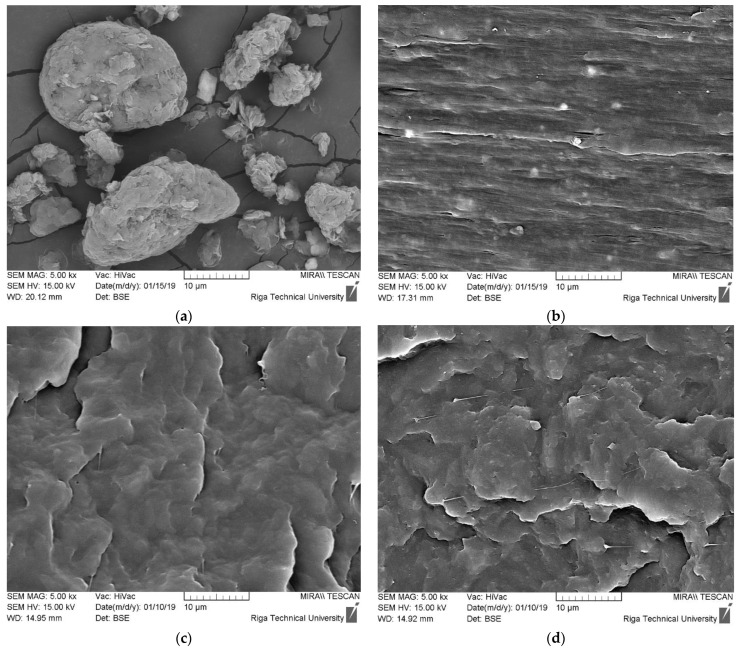

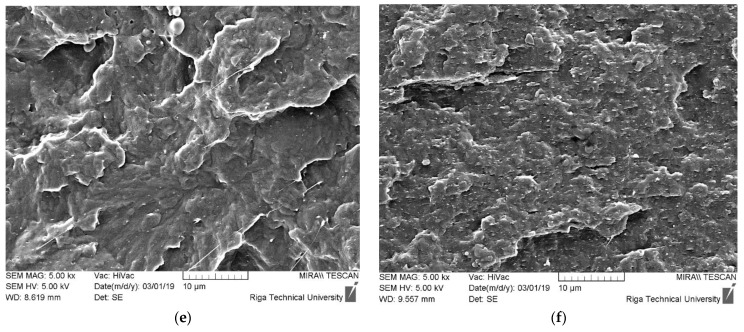
Surface morphology of C (**a**), M (**b**), and PP (**c**); its composites with 0.5%C and MAH (**d**), 0.5%M and MAH (**e**), and 5%M and MAH (**f**).

The surface morphology of C and M used for the development of PP nanocomposites is depicted in Figure 3. Before melt compounding with polymer matrix, C is in the form of large agglomerates with great variety in particle sizes. In Figure 3a, one can see that the agglomerates consist of a great number of plate-like C particles, which need to be separated during melt compounding. In the case of commercially marketed M (Figure 3b), one can observe that these clay particles are evenly distributed within the PP matrix, disregarding its high content (50 wt.%). Bright spots in the SEM images may be attributed to electron backscattering from the lateral surfaces of clay nanoparticles. In Figure 3c, the smooth surface morphology of a neat PP matrix is demonstrated.

The effect of nanoclay addition on the investigated melt compounded PP composites is demonstrated in Figure 3d–f. As one can see, regular distribution of the nanofiller within the PP matrix is observed, disregarding the type of the used particulate modifier (C or M) or its concentration in the polymer (0.5 wt.% or 5 wt.%). This testifies that the chosen processing conditions were sufficient to ensure regular distribution of both C and M within the used PP homopolymer matrix.

### 3.3. FTIR

**Figure 4:**
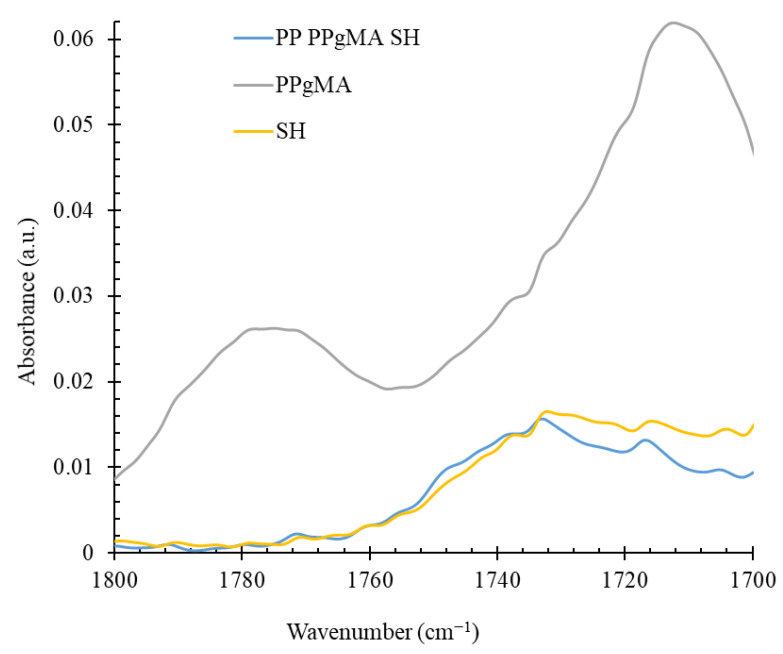
FTIR spectra of SH, PPgMA, and PPSH composite.

FTIR spectra in the region of absorption of carbonyl groups of SH, PPSH, and PPgMA are summarized in Figure 4. It has been well accepted that interaction between hydroxyl groups of lignocellulosic filler and maleic anhydride functional groups in PPgMA occurs via an esterification reaction leading to the formation of ester carbonyl groups. In Figure 4, it is demonstrated that absorption of the maleic anhydride groups grafted to PP occurs in the range between 1760 cm^−1^ and 1800 cm^−1^, with broad maxima from 1772 cm^−1^ to 1780 cm^−1^. After compounding with SH, absorption in this region is considerably decreased, whereas new signals have been observed in the range between 1744 cm^−1^ and 1746 cm^−1^. These signals may be attributed to the esterification reaction between free hydroxyl groups of SH and maleic anhydride functional groups of PPgMA.

### 3.4. Rheological Characterization

**Figure 5:**
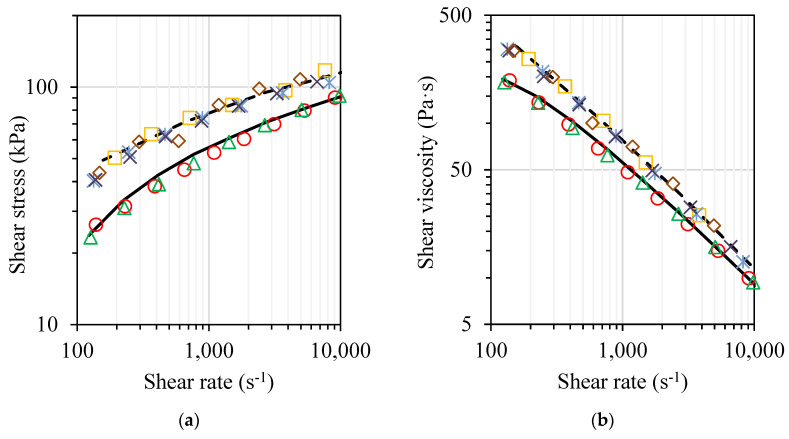

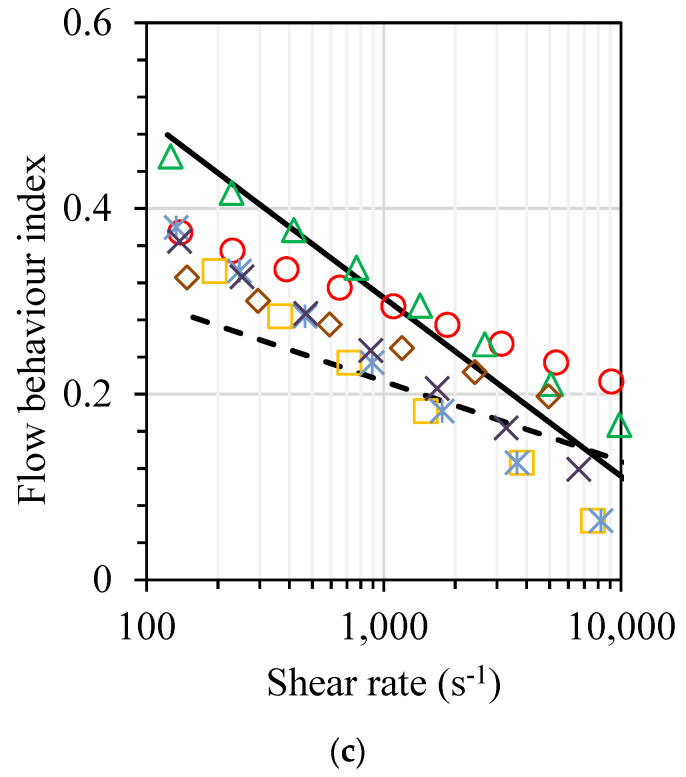
Shear stress (**a**), shear viscosity (**b**), and flow behaviour index (**c**) of PP (solid line), PP composite with SH (dashed line), PP nanocomposites with 5 wt.% of C (○) or M (Δ), PP hybrid composites with SH and 1 wt.% of C (□) or M (ж), and PP hybrid composites with SH and 3 wt.% of C (◊) or M (×) as functions of shear rate.

Figure 5 shows the shear rate dependence of viscosity, shear stress, as well as flow behaviour index for neat PP and PP composites, determined at 190 °C. The range of shear rates examined is characteristic for both polymer extrusion and injection moulding.

As depicted in Figure 5, in the investigated shear rate range, all the examined materials show a shear-thinning non-Newtonian behaviour caused by the disentanglement process and an increase in the average end-to-end distance of polymer chains as a result of shearing. Considerable shear stress and viscosity increase is observed along with the introduction of 40 wt.% of SH in the polymer matrix, whereas the effect of the addition of high surface area nanostructured nanoclay filler (C or M) is much lower.

However, it should be mentioned that at larger shear rate values approaching 10,000 s^−1^, viscosities of SH, C- and M-containing systems become rather similar. Evidently, at larger shear stress values, due to the orientation of the microphase lignocellulosic filler, its effect on the flow behaviour of the polymer composite melt becomes smaller. By analysing the viscosity and shear stresses of M- and C-containing systems, one can observe somewhat smaller data scatter. Hence, a more stable flow is observed in the case of clay masterbatch (M)-modified systems, especially in the case of hybrid composites with SH (see Figure 5a). By considering that in the certain shear rate region *τ*(*γ*·) and *η*(*γ*·) relationships are linear, the power law model is applied, allowing the determination of pseudoplasticity of the investigated systems. As demonstrated in Figure 5c, considerable differences in the pseudoplastic behaviour of the investigated systems are observed after the introduction of both SH and nanoclay containing reinforcing fillers. Upon the addition of micron-sized SH in the PP matrix melt, considerable changes in pseudoplastic behaviour are observed at lower shear rates range, whereas at high shear rates approaching 10,000 s^−1^, the value of flow behaviour index of the lignocellulosic filler-containing composite almost coincides with that of neat PP matrix. It is also worth mentioning that because of better dispersibility, the addition of the clay nanofiller in the form of masterbatch influences the pseudoplasticity of PP nanocomposites and PP hybrid composites considerably less in comparison with the systems containing neat nanoclay filler.

### 3.5. Thermal Properties

#### 3.5.1. Differential Scanning Calorimentry

DSC thermograms of PP, PPgMA, and their composites with SH, C, and M are reported in Supplementary Materials Figures S1–S3. A summary of DSC results for PP and PP composites modified with SH, C, and M is also reported in Table 2. It is found that PP modified with SH, C, and M demonstrate no considerable differences in melting behaviour. On first heating melting peak temperature *T_m_* of the PP matrix phase changes within the range of 167–169 °C, whereas after eliminating thermal prehistory on the second heating T_m_ range is lower −163–165 °C. Somewhat larger changes, fluctuating in the range from 36% to 53%, are observed for crystallinities *X_C_* of the melting PP phase. In general, on the introduction of either lignocellulosic flat microfibres or nanoclay particles, the crystallinity degree of the PP matrix is decreased, most probably because of the additive hindering formation of PP spherulites. However, during the cooling run crystallisation of the PP matrix is somewhat promoted as the cooling peak temperature of the polymer matrix phase is increased from 113 °C (PP) to 117 °C–119 °C (PP nanocomposites) and 116 °C–117 °C (the SH flat microfibre-reinforced and the hybrid composites). A somewhat greater increase in PP crystallisation peak temperature in the nanocomposites case evidently is associated with the prevalence of high-surface-area nanosized clay as nucleants. An increase in the *Xc* of PP with the incorporation of rigid fillers has also been reported by other authors [3,34].

#### 3.5.2. Thermogravimetric Analysis

TGA and TGA derivative thermograms of PP, PPgMA, and their composites with SH, C, and M are reported in Supplementary Materials Figures S4 and S5. Summary TGA results of PP and PP composites modified with SH, C, and M are also reported in Table 3. Initially, it should be mentioned that all the investigated compositions are sufficiently stable at the compounding temperatures used; according to the performed TGA experiments, less than 1% of the weight is lost at 220 °C. As expected, the SH-containing PP composite demonstrated the lowest thermal stability that was due to thermal degradation of thermally sensitive fractions of lignocellulose, particularly hemicelluloses, as described more in detail in numerous previous investigations, for example, [12,14]. Consequently, 5% of weight loss in PPSH composite is fixed at 271 °C, which is 1.5 times lower than for neat PP matrix (405 °C). Although the maximum degradation temperature *T_d_* of the composite after the introduction of SH does not change much (454 °C for PPgMA and 451 °C for PPSH), it should be mentioned that the remaining mass at 800 °C in the case of PPSH is considerably larger (11%) in comparison with PP (almost 0%). The thermal stability of the composite is increased with the addition of nanoclay in the system due to the ability of nanoclay to form combustible gases impermeable layer of char. As a result, the thermal stability of the investigated PP nanocomposites is considerably improved with the addition of nanoclay, especially M. It is important to mention that even a small amount of nanoclay (i.e., 0.5 wt.%) is sufficient to ensure a rise in thermal stability. Further increase in nanoclay filler content does not lead to a significant rise in thermal stability. At higher content of the nanoclay filler, thermal stability may even decrease due to the increased possibility of agglomeration of the nanofiller because of its high surface activity [34]. However, considering that according to SEM investigations, no notable agglomeration is observed for PP nanocomposites, a more plausible reason for somewhat reduced thermal stability is the presence of trace metal ions within clays. It has also been observed that the addition of nanoclay in the form of masterbatch ensured somewhat higher thermal stability in comparison with the systems containing pristine nanoclay. The addition of nanoclay in either form (C or M) is also effective in the case of the investigated hybrid composites. It is important to mention that in comparison with PPSH, a considerable increase of *T_50%_* and *T_75%_* is observed with the addition of clay nanofiller. Evidently, clay nanofiller, together with SH, promotes the development of a solid char layer. Consequently, hybrid composites demonstrate the highest *T_d_* values in comparison with all other investigated materials.

### 3.6. Mechanical Properties

#### 3.6.1. Flexural Test

**Figure 6:**
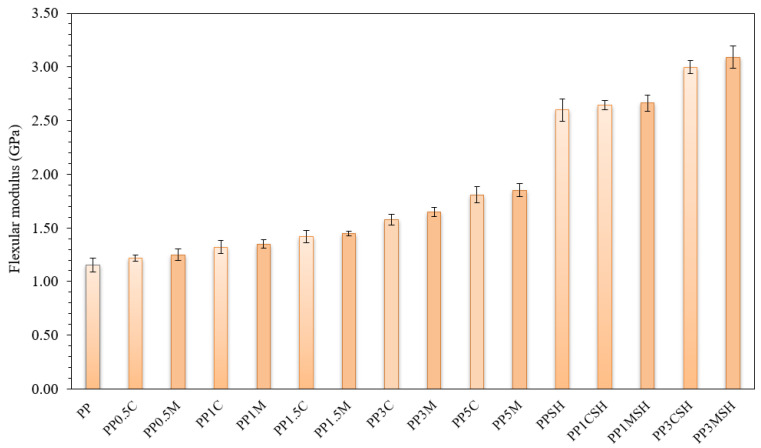
Flexural modulus of PP composites compared to neat PP; darker bars are related to the masterbatch-containing systems.

**Figure 7:**
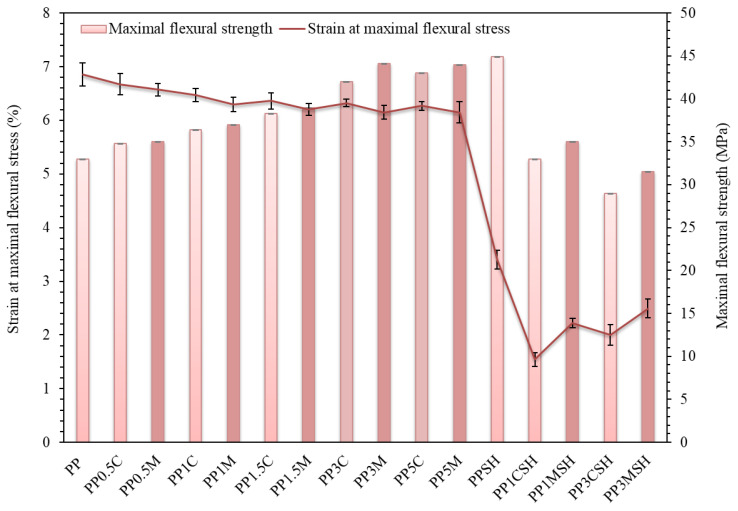
Strain at maximal flexural stress and maximal flexural strength of PP composites and neat PP; darker bars are related to the masterbatch-containing systems.

Analysis of the results of the flexural test is depicted in Figure 6 and Figure 7. The addition of either lignocellulosic flat microfibres or nanoclay fillers to PP results in increased stiffness and strength of the composites compared with neat PP. By considering nanoclay-modified polymer composites, a smooth increase in the modulus of elasticity and maximum flexural strength are observed by increasing the nanoclay content. Consequently, at the nanofiller content of 0.5 wt.% increase in flexural modulus is around 20%, while at maximum nanofiller content (5 wt.%) ca. 1.5-fold growth is observed. As expected, the modulus increase is larger in the case of clay masterbatch containing PP nanocomposites. It is worth noting that in the case of nanoclay-containing systems average reinforcing efficiency of the filler is 11% per 1 wt.% of the filler added, which is considerably more than in the case of lignocellulosic flat microfibre-containing systems for which ca. 3.0% increase per 1 wt.% of the added SH is observed. The introduction of the nanoclay filler in the PP matrix leads to a notable increase in maximal flexural stress, whereas strain at maximal flexural stress is slightly decreased, as depicted in Figure 7. However, it should be mentioned that all investigated PP nanocomposites, either with C or with M, were not broken during the test. Despite the high modulus values of hybrid composites (up to 3090 MPa for compatibilized PP composite with 40 wt.% SH and 3 wt.% M), unfortunately, an increase in brittleness accompanied by a reduction in maximal flexural stress and strain values is observed by rising the nanoclay content.

#### 3.6.2. Tensile Test

**Figure 8:**
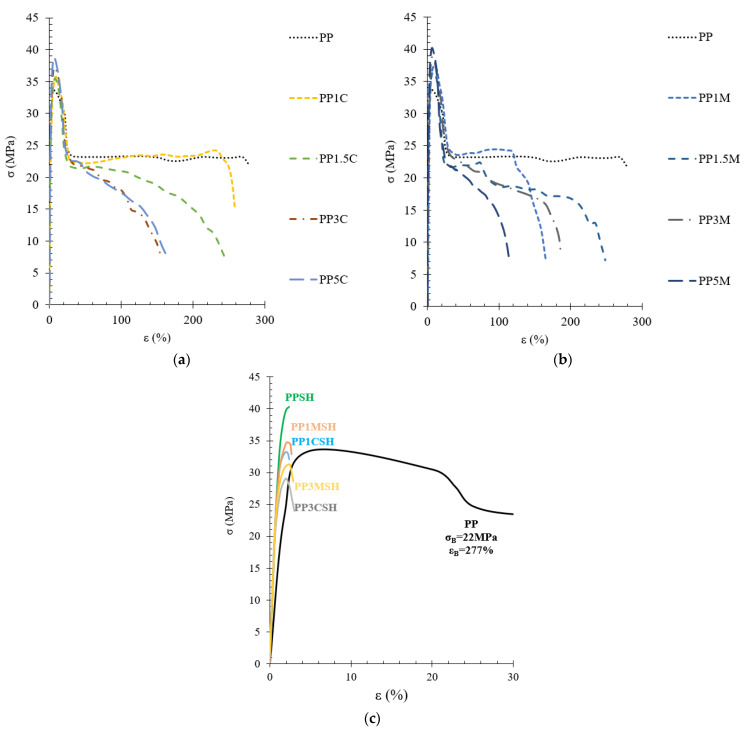
Tensile stress–strain diagrams of PP nanocomposites with C (**a**), M (**b**), and PP hybrid composites with SH and nanoclay (**c**).

Exemplary tensile stress–strain diagrams are depicted in Figure 8. As one can see, the addition of C and M caused an increase in yield strength but a certain decrease in the ultimate elongation of the investigated composites. A larger influence on the improvement of mechanical properties is observed in the case of masterbatch-containing systems, most probably due to better distribution of nanoclay within the polymer matrix. An increase in the nanoclay content in the polymer matrix above 1 wt.% considerably limits the reinforcing capability of the composite after yielding is achieved. However, this influence is small in comparison with that of SH addition. The addition of SH considerably increases the stiffness of the composite, as clearly shown in Figure 8c from the considerably raised slope of the *σ-ε* curves. Unfortunately, stiffness increase occurs, greatly compromising the deformability of the composites.

**Figure 9:**
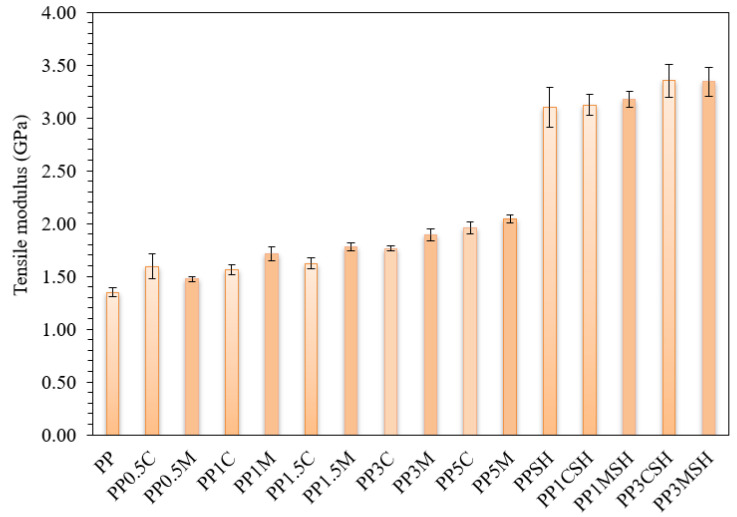
Tensile modulus of PP composites compared to neat PP; darker bars are related to the masterbatch-containing systems.

**Figure 10:**
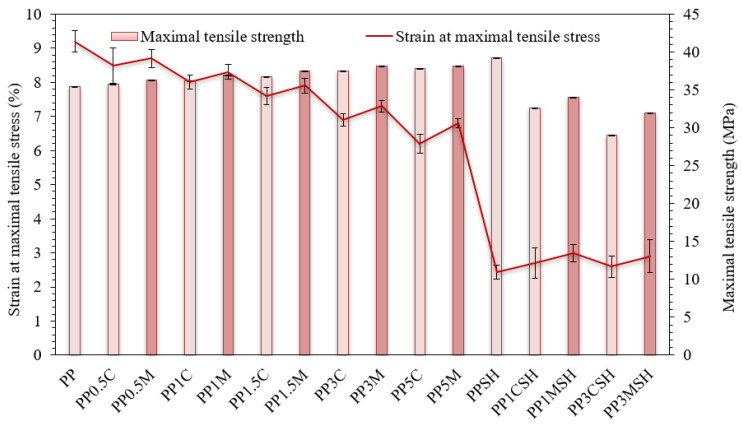
Strain at maximal tensile stress and maximal tensile strength of PP-modified composites and neat PP; darker bars are related to the masterbatch-containing systems.

A more detailed analysis of the results of the tensile test is depicted in Figure 9 and Figure 10. Tensile modulus, as well as the maximal tensile stress and strain values, are analysed. It is worth mentioning that in the case of the nanoclay-reinforced binary composites, the corresponding stress–strain values are attributed to the upper yield point, whereas in the case of hybrid composites, the corresponding stress–strain values are attributed to the fracture point. In general, the tensile behaviour of the investigated PP composites has similar trends to flexural behaviour. Consequently, the addition of only nanoclay results in increased modulus and strength of the nanocomposite by maintaining remarkable ultimate deformation values (even up to 150% at the highest nanofiller content), whereas the addition of nanoclay nanofiller to SH-containing PP composites further increases it brittleness (Figure 9 and Figure 10).

## 4. Conclusions

In this research, the efficiency of modification of PP by SH (40 wt.%), C, and M (1 and 3 wt.%) in the presence of PPgMA (3 wt.%) compatibiliser is demonstrated. The following results have been obtained:

(1) The addition of the nanoclay fillers does not considerably reduce melt viscosities of PP nanocomposites as well as PP hybrid composites with SH because of the regular dispersion of C or M particles within the polymer matrix, as demonstrated by SEM analysis;
(2) Increased modulus of elasticity of the nanoclay-modified composites is a result of the reinforcement effect of high-aspect-ratio plate-like clay nanoparticles, oriented in the direction of the flow, as well as the perfection of the crystalline structure of the polymer matrix, as demonstrated by the increase in melting peak temperature of PP crystalline phase;
(3) Increased modulus of elasticity of hybrid composites is due to the reinforcement effect of high-aspect-ratio nanoclay particles and isodiametric SH particles (aspect ratio within the range 2–6) oriented in the direction of the flow;
(4) Reduced strength of the investigated nano- and hybrid composites is a result of the constraint in deformability (ultimate elongation values) of PP matrix introduced in the presence of rigid fillers;
(5) Improved thermal stability of PP nano- and hybrid composites is attributed to the thermal barrier effect of plate-like nanoclay, increasing the 5% weight loss temperature (*T_5%_*) of PP nanocomposites on the one hand and promoting more intense development of impermeable char layer related to increased maximal degradation temperature (*T_d_*) of PP hybrid composites on the other hand.

Consequently, the developed hybrid composites can be regarded as environmentally friendly alternative materials for the development of injection-moulded products requiring increased thermal stability and stiffness.

## Figures and Tables

**Table 1:** Codes and composition of the composites.

Code	PP (wt.%)	PPgMA (wt.%)	C (wt.%)	M (wt.%)	SH (wt.%)
PP	100	-	-	-	-
PPSH	56.7	3.3	-	-	40
PP0.5C	96.2	3.3	0.5	-	40
PP0.5M	95.7	3.3	-	1	40
PP1C	95.7	3.3	1	-	40
PP1M	94.7	3.3	-	2	40
PP1.5C	95.2	3.3	1.5	-	40
PP1.5M	93.7	3.3	-	3	40
PP 3C	93.7	3.3	3	-	40
PP 3M	90.7	3.3	-	6	40
PP5C	91.7	3.3	5	-	40
PP 1C SH	55.7	3.3	1	-	40
PP1M SH	54.7	3.3	-	2	40
PP3C SH	53.7	3.3	3	-	40
PP3M SH	50.7	3.3	-	6	40

**Table 2:** Calorimetric properties of the investigated PP and PP composites.

Sample	Heating Run 1		Heating Run 2		Cooling Run	
	*T_m_* (°C)	*X_C_* (%)	*T_m_* (°C)	*X_C_* (%)	*T_m_* (°C)	*X_C_* (%)
PP	167	45	162	51	113	52
PPgMA	135	61	132	63	99	65
PPSH	167	42	164	43	117	53
PP1.5C	170	36	164	42	118	51
PP1.5M	168	42	164	46	118	50
PP1C	169	46	163	51	118	49
PP1M	169	40	164	46	118	47
PP1.5C	168	44	165	49	118	52
PP1.5M	168	43	163	47	117	51
PP3C	168	45	165	49	117	50
PP3M	169	42	165	42	119	53
PP5C	169	41	165	42	118	51
PP5M	169	42	166	45	117	51
PP1CSH	168	36	165	40	116	45
PP1MSH	166	40	164	41	117	50
PP3CSH	167	40	165	49	117	51
PP3MSH	168	40	166	45	116	52

**Table 3:** Thermogravimetric properties of the investigated PP and PP composites.

Test Specimen Identification	Weight Loss Temperatures				Maximum Degradation Temperature
	*T_5%_* (°C)	*T_10%_* (°C)	*T_50%_* (°C)	*T_75%_* (°C)	*T_d_* (°C)
PP	405	422	453	461	453
PPgMA	323	385	444	457	454
SH	271	294	434	458	451
PPSH	271	294	434	472	451
C	303	328	-	-	404
M	363	405	437	433	436
PP0.5C	426	433	446	449	449
PP0.5M	429	434	445	449	447
PP1C	424	430	439	446	440
PP1M	424	430	440	447	441
PP1.5C	423	428	437	453	440
PP1.5M	426	431	443	445	446
PP3C	422	428	438	442	440
PP3M	426	432	443	451	446
PP5C	421	428	436	435	442
PP5M	419	427	435	433	440
PP1CSH	273	295	457	474	465
PP1MSH	267	291	458	475	467
PP3CSH	266	291	462	480	474
PP3MSH	272	293	464	482	474

## Data Availability

The data presented in this study are available on request from the corresponding author.

## Supplementary Materials

The following supporting information can be downloaded at: https://www.mdpi.com/article/10.3390/polym14204332/s1, Figure S1: DSC heating run 1 of PP, PPgMA, and its composites with SH, C, and M, Figure S2: DSC cooling run of PP, PPgMA and its composites with SH, C, and M, Figure S3: DSC heating run 2 of PP, PPgMA and its composites with SH, C, and M, Figure S4: TGA thermograms of PP, PPgMA and its composites with SH, C, and M, Figure S5: TGA derivative thermograms of PP, PPgMA and its composites with SH, C, and M.
