# Supplementary material for: Characterisation of Nanoclay and Spelt Husk Microfiller-Modified Polypropylene Composites
Source: Polymers (Basel). 2022 Oct 14;14(20):4332. doi: 10.3390/polym14204332 (PMC9609350; doi:10.3390/polym14204332)
Supplement: Supplementary file 1 [file polymers-14-04332-s001.zip › polymers-1932431-supplementary.pdf]

Supplementary Materials

Characterisation of Nanoclay and Spelt Husk Microfiller-Modified Polypropylene Composites

Madara Žiganova [1,*], Remo Merijs-Meri [1], Jānis Zicāns [1], Tatjana Ivanova [1], Ivan Bochkov [1], Mārtiņš Kalniņš [1], Andrzej K. Błędzki [2] and Paulius P. Danilovas [3]

[1] Institute of Polymer Materials, Faculty of Materials Science and Applied Chemistry, Riga Technical University, 3 Paula Valdena Street, LV-1048 Riga, Latvia
[2] Institute of Materials Science, West Pomeranian University of Technology, Department of Materials Engineering, 19 Piastów Avenue, 70310 Szczecin, Poland
[3] Polymer Competence Center, Gradauciznos k. 7, LT-60430 Raseiniu r., Lithuania
* Correspondence: madara.ziganova@rtu.lv; Tel.: +37-167-089-2525

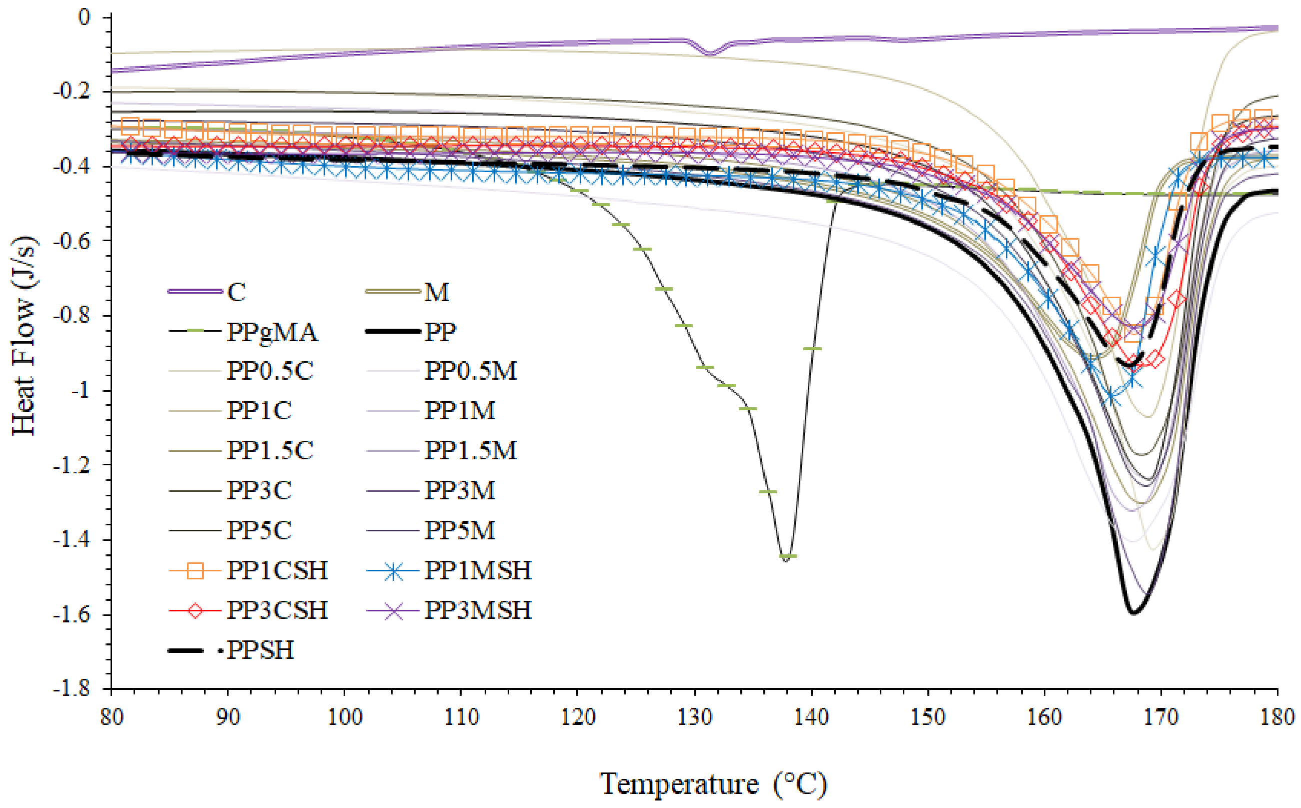

Figure S1. DSC heating run 1 of PP, PPgMA, and its composites with SH, C, and M.

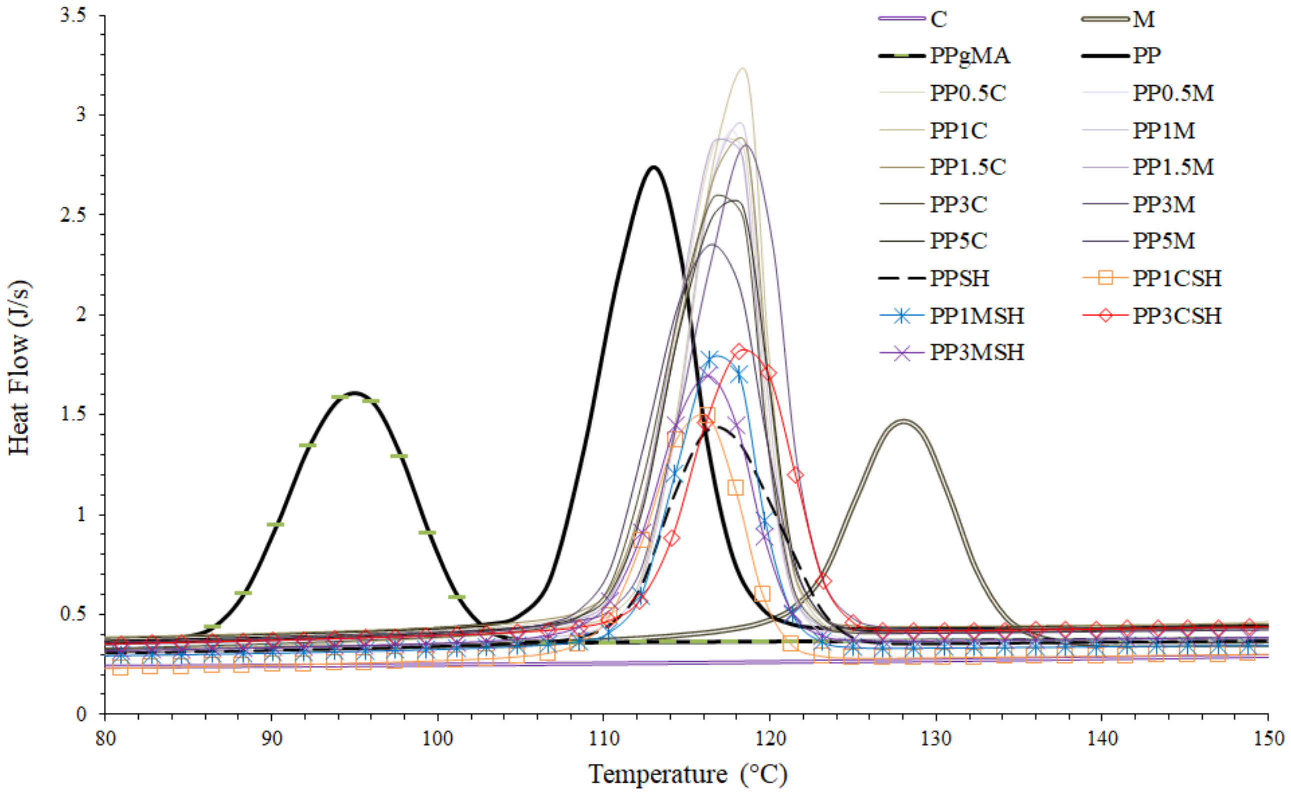

Figure S2. DSC cooling run of PP, PPgMA and its composites with SH, C, and M.

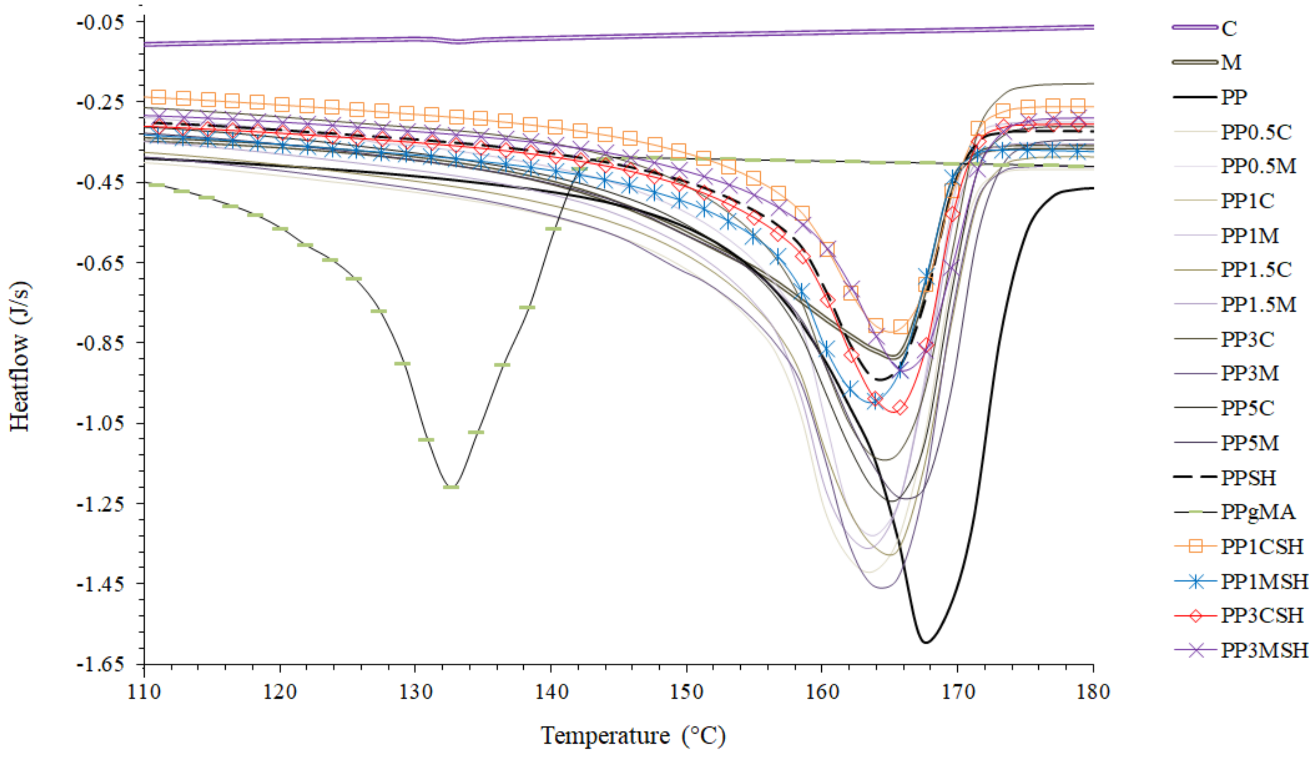

Figure S3. DSC heating run 2 of PP, PPgMA and its composites with SH, C, and M.

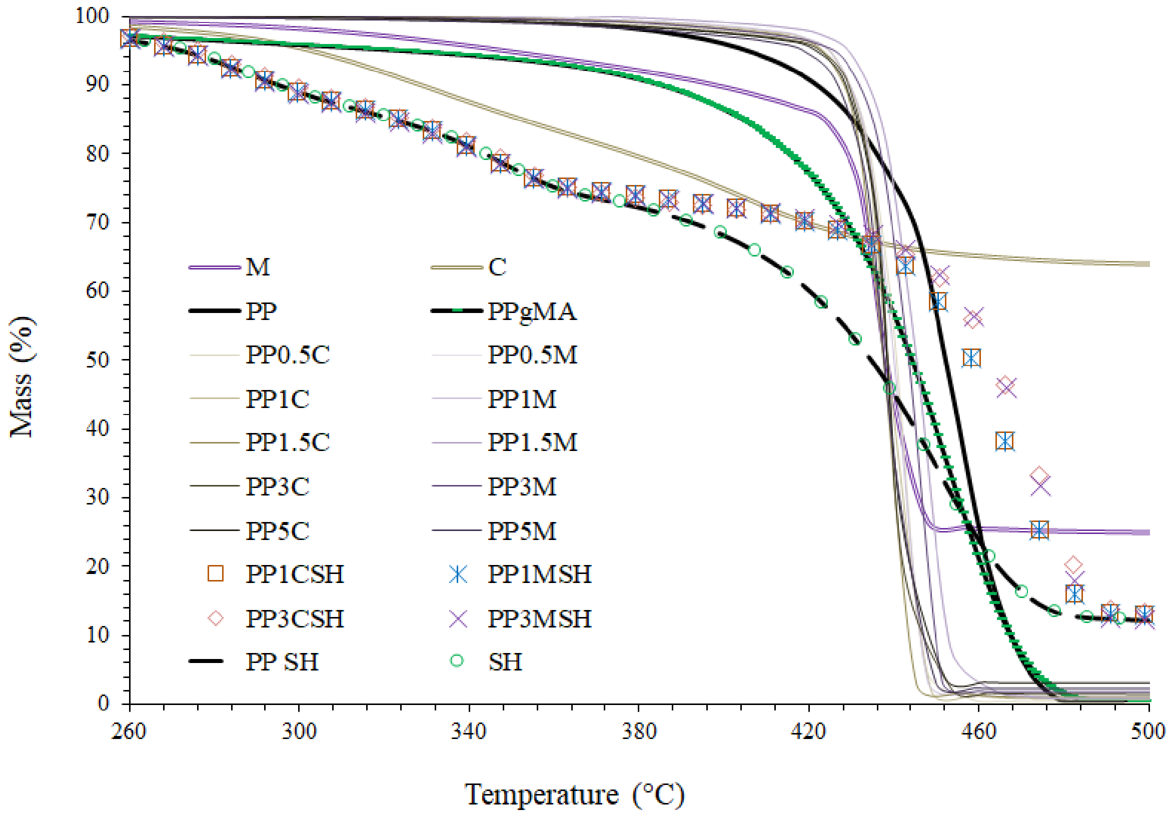

Figure S4. TGA thermograms of PP, PPgMA and its composites with SH, C, and M.

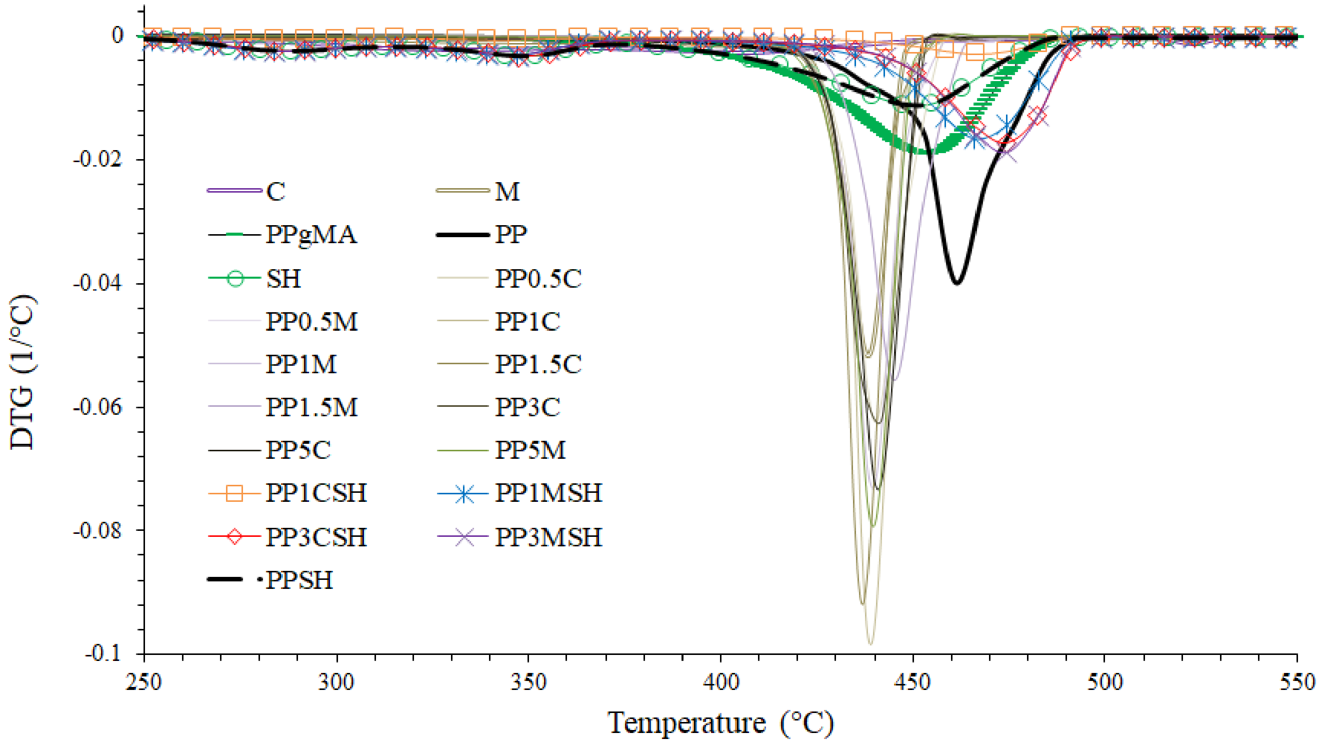

Figure S5. TGA derivative thermograms of PP, PPgMA and its composites with SH, C, and M.
